# Gamma rays excited radioluminescence tomographic imaging

**DOI:** 10.1186/s12938-018-0480-x

**Published:** 2018-04-24

**Authors:** Xuanxuan Zhang, Shouping Zhu, Yang Li, Yonghua Zhan, Xueli Chen, Fei Kang, Jing Wang, Xu Cao

**Affiliations:** 1 Engineering Research Center of Molecular and Neuro Imaging of the Ministry of Education & School of Life Science and Technology, Xidian University, Xi’an, 710071 Shaanxi China; 2 Department of Nuclear Medicine, Xijing Hospital, Fourth Military Medical University, Xi’an, 710032 Shaanxi China

**Keywords:** Radioluminescence imaging, Tomography, Diffusion equation, Image reconstruction

## Abstract

**Background:**

Radionuclide-excited luminescence imaging is an optical radionuclide imaging strategy to reveal the distributions of radioluminescent nanophosphors (RLNPs) inside small animals, which uses radioluminescence emitted from RLNPs when excited by high energy rays such as gamma rays generated during the decay of radiotracers used in clinical nuclear medicine imaging. Currently, there is no report of tomographic imaging based on radioluminescence.

**Methods:**

In this paper, we proposed a gamma rays excited radioluminescence tomography (GRLT) to reveal three-dimensional distributions of RLNPs inside a small animal using radioluminescence through image reconstruction from surface measurements of radioluminescent photons using an inverse algorithm. The diffusion equation was employed to model propagations of radioluminescent photons in biological tissues with highly scattering and low absorption characteristics.

**Results:**

Phantom and artificial source-implanted mouse model experiments were employed to test the feasibility of GRLT, and the results demonstrated that the ability of GRLT to reveal the distribution of RLNPs such as Gd_2_O_2_S:Tb using the radioluminescent signals when excited by gamma rays produced from ^99m^Tc.

**Conclusions:**

With the emerging of targeted RLNPs, GRLT can provide new possibilities for in vivo and noninvasive examination of biological processes at cellular levels. Especially, combining with Cerenkov luminescence imaging, GRLT can achieve dual molecular information of RLNPs and nuclides using single optical imaging technology.

## Background

Cerenkov luminescence imaging (CLI) can provide distributions of radiotracers by traditional optical imaging technologies [1–3]. It has been used for measuring tumor burden after chemotherapy administration [4, 5], surgical resection [6, 7], and even clinical studies [8–10]. A tomographic imaging based on Cerenkov luminescence has also been proposed to obtain three dimensional distributions of radiotracers [11–16]. However, CLI is limited by its relatively weak optical intensity and poor penetration [7, 9].

A recently emerged optical radionuclide imaging technique, radionuclide-excited luminescence imaging (RELI), is a potential strategy for improving optical intensity, which uses the radioluminescent signals emitted from radioluminescent nanophosphors (RLNPs) when excited by high energy rays such as gamma rays and beta rays generated during the decay of radionuclides [17–19]. RELI can surpass the weakness and penetration limitation of CLI and provide a new optical approach for imaging radiotracers used in PET [20]. As an internal activatable molecular imaging, RELI based on europium oxide nanophosphor has been performed to detect tumor lesions [21]. The results demonstrated that RELI can provide strong optical signals with high signal-to-background ratios in early and small tumor detection. Investigators have also performed in vitro and in vivo imaging of ^99m^Tc using RELI with scintillating crystals [22]. Similarly, with an ingenious design using scintillator and steel plate, β particles excited RELI was evaluated in imaging of amelanotic and melanotic tumors [23]. Combined with microscopic imaging technology, a radioluminescence microscopy was proposed to visualize ^18^F-FDG uptake in single living cell [24]. A fiber-optic RELI system with a scintillator was developed for imaging ^18^F-FDG and fluorescent glucose probes from in vitro atherosclerotic plaques [25].

Similarly, X-ray was also used to excite RLNPs in X-ray luminescence imaging (XLI), and the emitted X-ray luminescence was acquired by CCD camera [26]. Tomographic imaging based on X-ray luminescence irradiated by a selective X-ray beam scanning scheme was also reported by Carpenter [27–29]. The proposed x-ray luminescence computed tomography (XLCT) can simultaneously provide 3D molecular and anatomical information. To reduce acquisition time, a focused micrometers size X-ray spot using polycapillary lens [30, 31] and cone beam X-ray [32, 33] were used to excite the imaged object.

Due to the excellent penetration of gamma rays in biological tissues, there is no depth limitation on excitation of RLNPs. Besides, optical intensity of RELI is much higher than that of CLI when radionuclide such as ^99m^Tc is used as the excitation source. These advantages make it possible for RELI to imaging deep lesions for biological applications. But this photographic imaging of RELI can only provide two-dimensional images of radioluminescent signals on the surface of a small animal, which is difficult to reveal distributions of RLNPs deep in the small animal as a result of high scattering of photons in biological tissues.

In this paper, gamma rays excited radioluminescence tomography (GRLT) is proposed to reconstruct three-dimensional distribution of RLNPs inside a small animal using optical measurements of radioluminescence. This internal excitation by radionuclides for GRLT can markedly decrease noises such as auto-fluorescence or leakage of excitation lights in conventional fluorescence imaging, and result in greater signal-to-background ratios. To obtain a tomographic imaging based on radioluminescence, the diffusion equation was employed to model propagations of radioluminescent photons in biological tissues with highly scattering and low absorption characteristics [34–36]. A tangential planar approach, the Kirchhoff approximation, was used to achieve an analytical formulation of the Green’s functions of the diffusion equation [37]. After the discretization of imaged medium, a linear equation can be established to describe the relationship between the flux of the radioluminescent source at internal voxels of the grid and the boundary measurements of radioluminescent photons. Phantom and artificial source-implanted mouse model experiments were implemented to validate the feasibility of GRLT.

## Methods

In this work, the used radionuclide ^99m^Tc was obtained from the Department of Nuclear Medicine, Xijing Hospital, the Fourth Military Medical University (FMMU), which produces pure gamma rays with 140 keV. The used RLNPs was terbium doped Gd_2_O_2_S (Gd_2_O_2_S:Tb), which was purchased from Shanghai Keyan Phosphor Technology Co. Ltd with the mean size of about 100 nm. Gd_2_O_2_S:Tb is a common used phosphor material in x-rays photography, which can convert x-rays to visual lights. Nowadays, water-soluble Gd_2_O_2_S:Tb has been used in small animal imaging [38, 39].

**Fig. 1:**
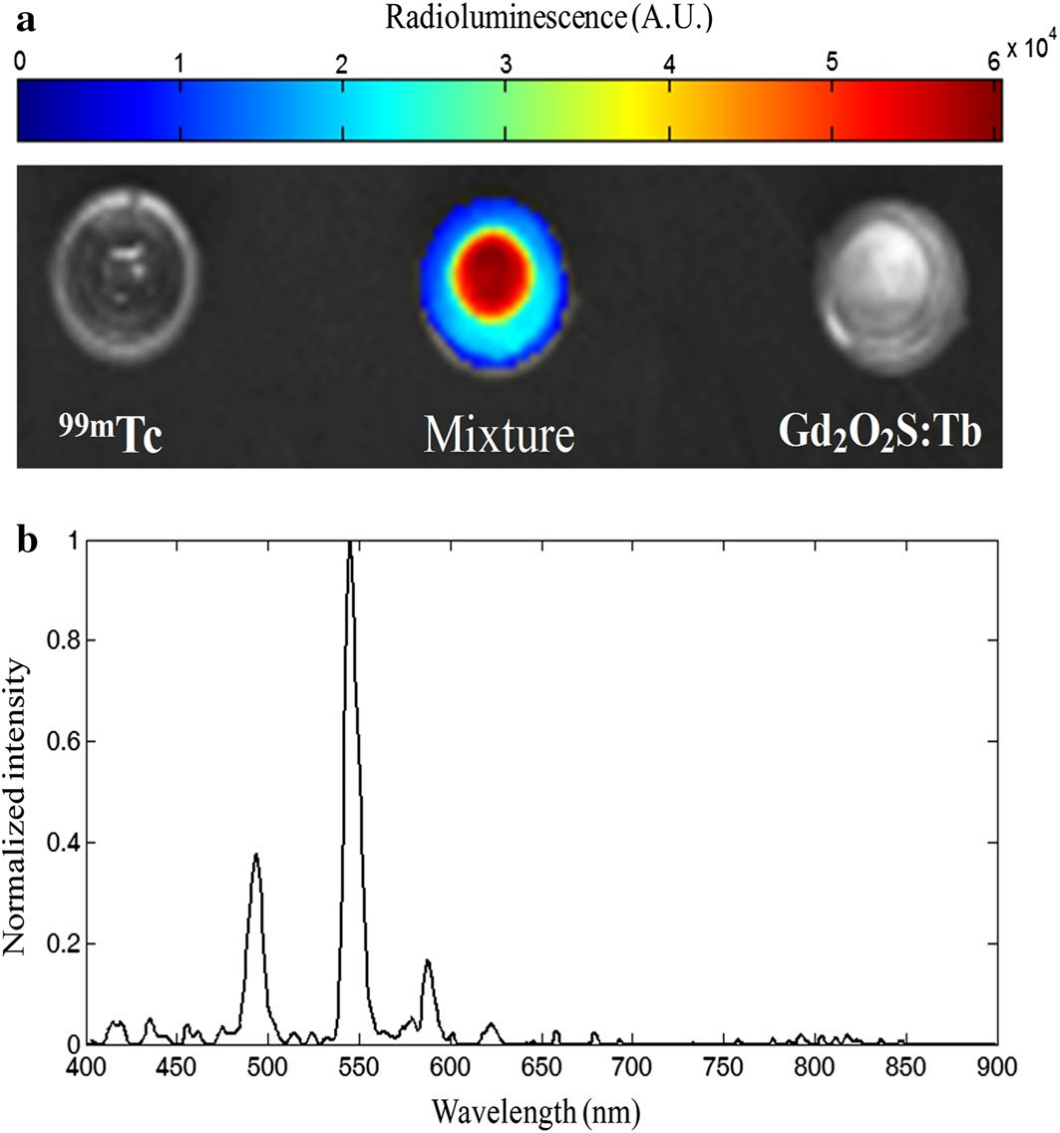
**a** Optical measurements of ^99m^Tc, the mixture and Gd_2_O_2_S:Tb. **b** Radioluminescence spectrum of Gd_2_O_2_S:Tb excited by gamma rays (140 keV) produced from ^99m^Tc

To illustrate the origin of radioluminescent signals, we investigated three situations of 100 µL Na^99m^TcO_4_ with an activity of 7.4 MBq (200 µCi), Gd_2_O_2_S:Tb with 10 mg, and a mixture of them. Each of the sample was put into a small plastic container, and imaged by a homemade optical imaging system configured by an EMCCD (iXon3 888, Andor) camera coupled with a focus lens (25 mm f/0.95 s, Schneider) simultaneously. The exposure time and EM gain were set to 5 s and 100, respectively. In Fig. 1a, the left, middle and right are the single ^99m^Tc, the mixture and the single Gd_2_O_2_S:Tb, respectively. There were strong radioluminescent signals from the mixture, but no signals captured for the single ^99m^Tc and Gd_2_O_2_S:Tb. It is known that ^99m^Tc alone emits a weak radioluminescence light and can be detected using IVIS imaging system [40]. The low activity of ^99m^Tc and short exposure time are the reasons for non- captured signals from ^99m^Tc.

This implies the radioluminescent signal is mainly produced when Gd_2_O_2_S:Tb is excited by gamma rays generated during the decay of ^99m^Tc. Furthermore, the spectrum of radioluminescence was measured using a fluorescence spectrophotometer (F-4500, Hitachi, Japan). Because the mixture itself can emit optical signals without external excitation, the lamp of fluorescence spectrometer was close when measuring the spectrum. The spectrogram of the radioluminescence of the mixture is shown as in Fig. 1b, and there are three peaks at 490, 545 and 585 nm.

For GRLT radionuclides inside a small animal are used as the internal excitation source, whose distributions are very complex and varying with time. To simplify the model of GRLT, here we just consider the distributions of emitted radioluminescence. To obtain a tomographic imaging based on radioluminescence, the diffusion equation is employed to model propagations of radioluminescent photons in biological tissues with high scattering and low absorption characteristics [34–36]:

1 $$\left\{ \begin{aligned} { - }\nabla \cdot [D(r)\nabla \varPhi (r)] + \mu_{a} (r)\varPhi (r) = X(r)\begin{array}{*{20}c} {} & {\begin{array}{*{20}c} {} & {} \\ \end{array} } & {r \in \varOmega } \\ \end{array} \hfill \\ \varPhi (r) + 2AD(r)\nabla \varPhi (r) \cdot \hat{n} = 0\begin{array}{*{20}c} {\begin{array}{*{20}c} {\begin{array}{*{20}c} {\begin{array}{*{20}c} {\begin{array}{*{20}c} {} & {} \\ \end{array} } & {} \\ \end{array} } & {} \\ \end{array} } & {} \\ \end{array} } & {\quad r \in \partial \varOmega } \\ \end{array} \hfill \\ \end{aligned} \right.$$

where Φ(*r*) is the flux density of radioluminescence, *X*(*r*) denotes the unknown radioluminescent yield, *µ*_*a*_ is the absorption coefficient, *D* = − 1/[3(*μ*_*a*_ + *μ*_*s*_′)] is the diffusion coefficient, and *μ*_*s*_′ is the reduced scattering coefficient. Ω and ∂Ω represent the regions of the object and its boundary, respectively. *A* is a boundary constant representing the refractive index mismatched between tissues and the surrounding medium. Here a tangential planar approach named Kirchhoff approximation can be used to achieve an analytical formulation of the Green’s functions of the diffusion equation [37]. After the discretization of the imaged medium, a linear equation can be established to describe the relationship between the flux of the radioluminescent source at internal voxels of the grid and the boundary measurements of radioluminescent photons:

2 $$WX = \varPhi$$

where *W* is the weight matrix mapping the unknown radioluminescent yield *X* into the measurable boundary light intensity Φ.

The inverse problem of GRLT is to solve Eq. (2), which is an ill-posed problem. An effective approach to overcome this ill-posedness is to reduce the number of unknowns by using a priori information about permissible source regions [41–43]. In this work, we use the observation vector *u* = *W*^T^Φ as a good local approximation to the unknown radioluminescent yield *X*. Based on the sparsity of GRLT, if the sparsity level of *X* is *N*, we can chose *N* biggest coordinates of the observation vector *u* as a permissible source region [44]. Finally, a traditional Tikhonov regularization is employed to solve Eq. (2) with a reduced solving domain decided by the permissible source region.

## Results and discussion

**Fig. 2:**
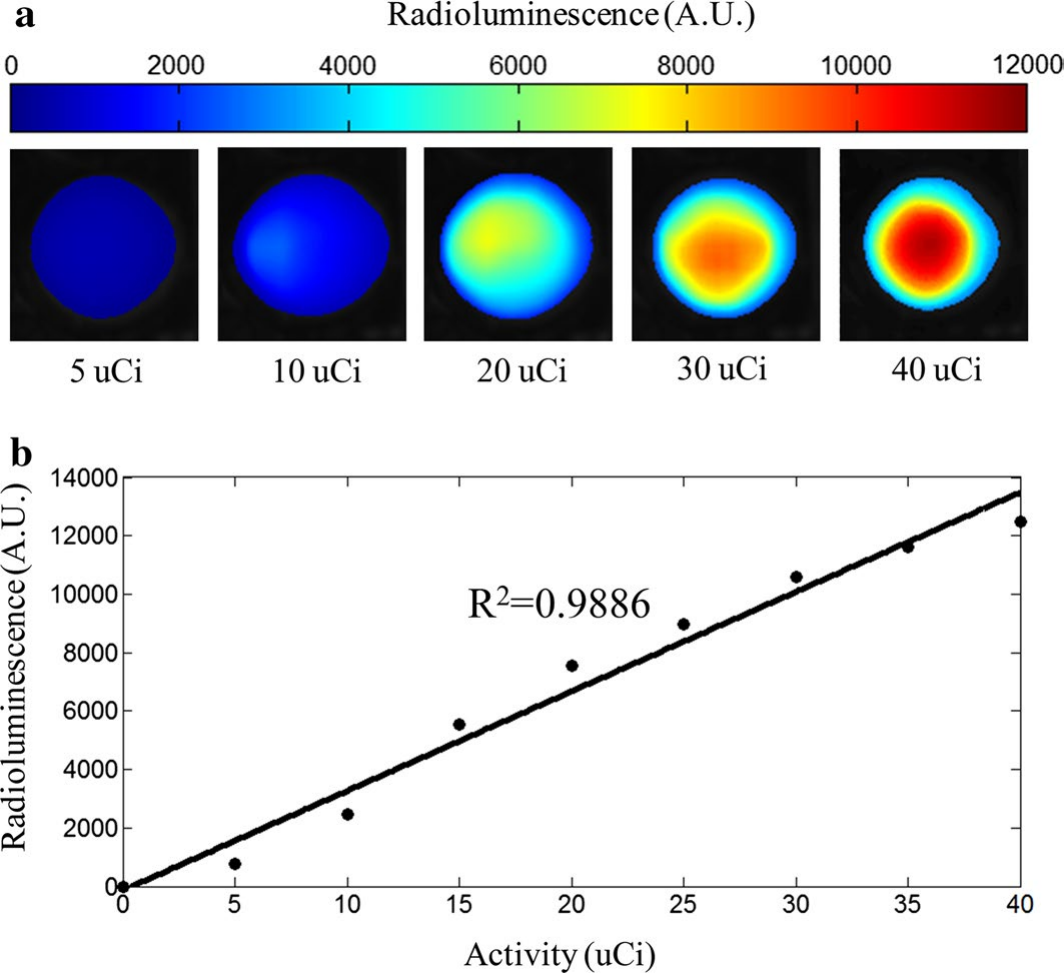
**a** Radioluminescence images when excited by ^99m^Tc with different activities. **b** Quantitative curve of the radioluminescence images

It is clear that the intensity of radioluminescent signal is related to the activity of radionuclide and the mass of RLNPs. RELI images for the mixtures of Gd_2_O_2_S:Tb and ^99m^Tc with different activities was captured and shown in Fig. 2a after a *12*  * 12 median filtering. The intensity of radioluminescent signal is increase with the activity of ^99m^Tc. The quantitative analysis of RELI images in Fig. 2a was obtained by calculated the mean intensity in region of interesting (ROI), and was shown in Fig. 2b. The intensity of radioluminescent signal linearly correlates with radioactivity of ^99m^Tc.

**Fig. 3:**
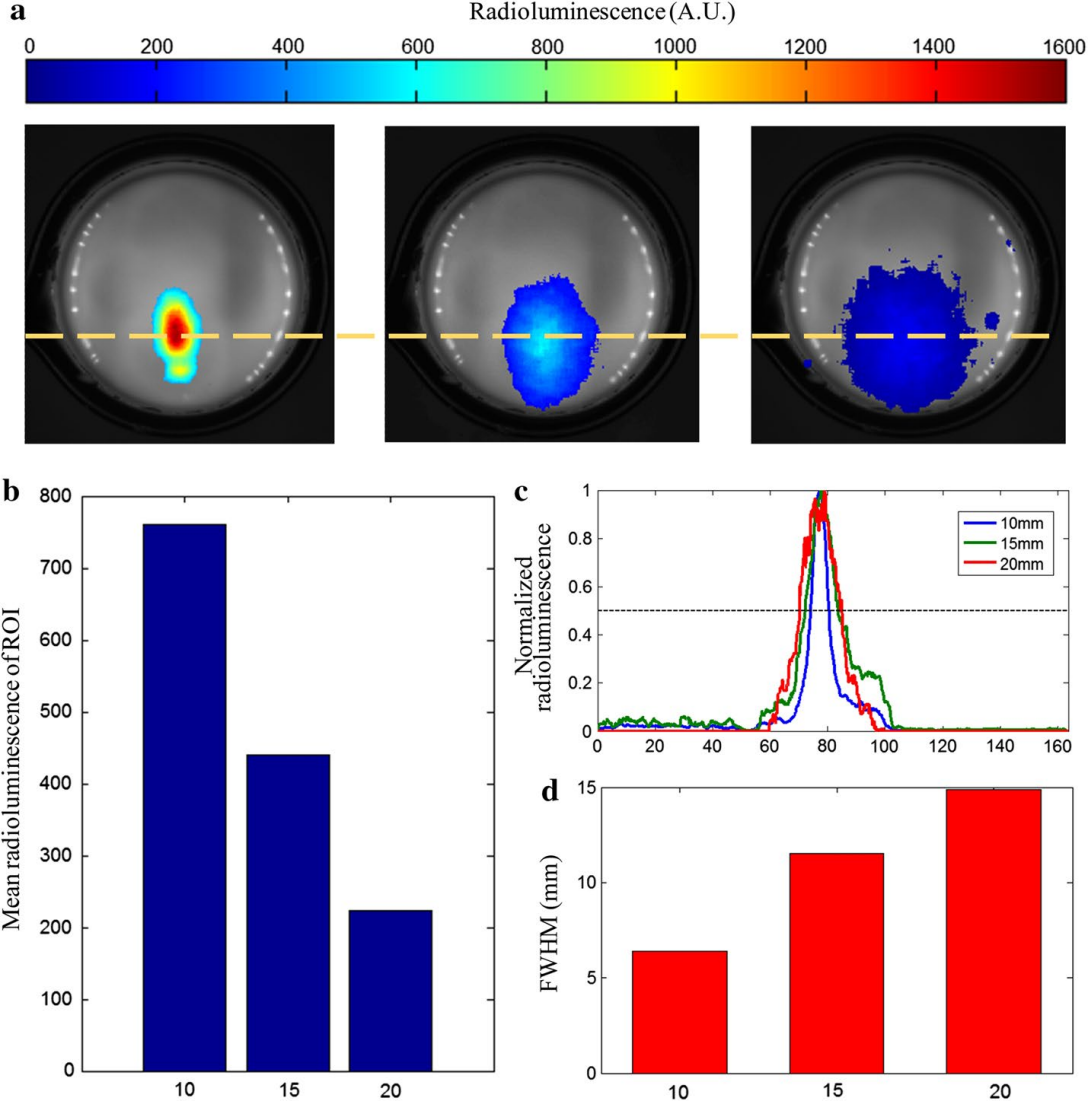
Penetration depth of radioluminescent signals. **a** RELI images fused with white images for different depths of radioluminescent target. **b** Quantitative analysis of radioluminescent signals for different depth. **c** Profiles of RLI images for different depths acquired along horizontal line defined in **a**. **d** Full width at half maximum of RELI images at different depths

To test the penetration depth of radioluminescent signals, a glass cup of 40 mm in height and 20 mm in diameter filled with 1% intralipid solution was employed as a physical phantom. Absorption and reduced scattering coefficients for the phantom are 0.002 and 1 mm^−1^, respectively, which are similar with those of biological tissues. A small glass tube filled with Na^99m^TcO_4_ with an activity of 3.7 MBq (100 µCi) and 10 mg Gd_2_O_2_S:Tb was used as the radioluminescent target, and fasten on the bottom of the glass cup. RELI images were captured when different volumes of intralipid solution were added into the glass cup to mimic different depths of the radioluminescent target, and results are summarized as Fig. 3. Radioluminescent signals decrease observably with depths of radioluminescent target as shown in RELI images (Fig. 3a). The quantitative analysis of radioluminescent signals is show in Fig. 3b by mean radioluminescence of ROI. The strong scattering of turbid medium severely degrades the resolution of RELI images with increase of depths as shown in Fig. 3c, d.

Phantom and artificial source-implanted mouse model experiments were implemented to validate the feasibility of GRLT. In phantom experiment, a polyoxymethylene made semi-cylinder with 20 mm in diameter and 30 mm in height was used as the phantom to mimic biological tissues. The absorption and scattering coefficients of the phantom were 0.002 and 1 mm^−1^, respectively. The phantom had a hole drilled along the axial direction with the depth of about 6 mm. The mixture of 100 µL Na^99m^TcO_4_ with an activity 3.7 MBq (100 µCi) and 10 mg Gd_2_O_2_S:Tb was put into a transparent glass capillary tube with an inner diameter of 2 mm and an outer diameter of 3 mm. Then the glass capillary tube to mimic the radioluminescent target was put into the hole of the semi-cylinder phantom.

**Fig. 4:**
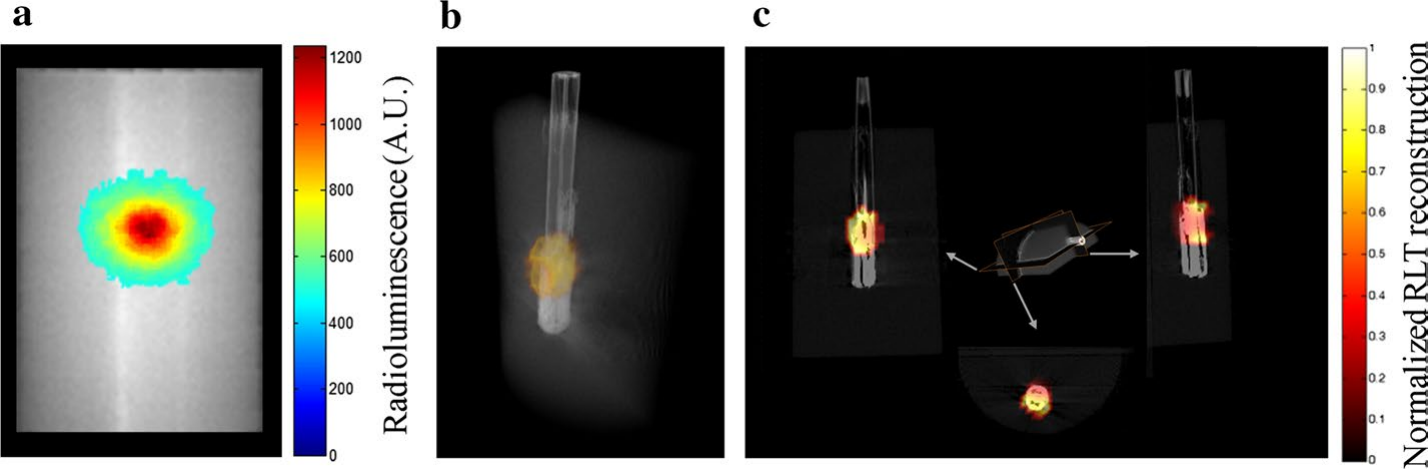
Results for the phantom experiment. **a** Radioluminescent image of the phantom. **b** 3D fusion image of CT and reconstructed radioluminescence signals. **c** Transversal, coronal and sagittal slices of reconstructed radioluminescence signals overlaid on corresponding CT slices

Radioluminescent signals were captured by our homemade optical imaging system with exposure time of 5 s for EMCCD camera, and the captured radioluminescent image was shown in Fig. 4a. Subsequently, micro-CT scans of the phantom were performed (50 kVp, 1.0 mA, 360 views with 1° interval). Filtered back-projection method was used to reconstruct micro-CT data of the phantom, which was used to extract the phantom surface for forward modal calculation of GRLT.

The reconstructed images fused with CT images are shown in Fig. 4b, c, which includes 3D volume rendering and 2D sections of the phantom reconstruction. The location of radioluminescent target reconstructed by GRLT visually matches that in CT image, which can accurately locate the radioluminescent target. This phantom result illustrates that GRLT can accurately obtain three-dimensional distributions of RLNPs when excited by gamma rays.

**Fig. 5:**
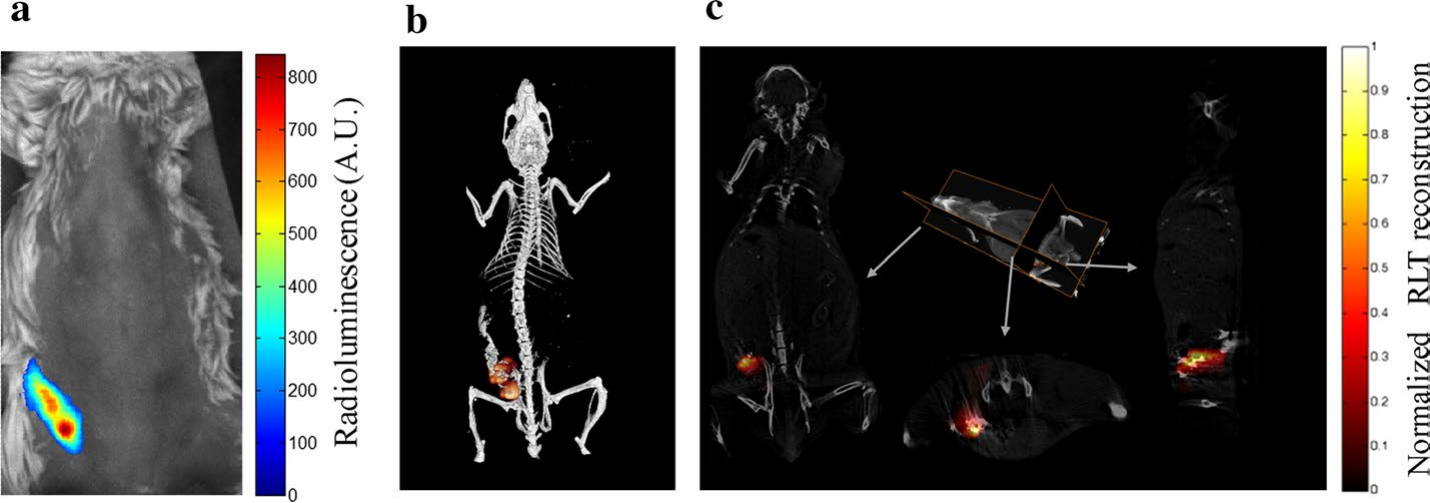
Results for the supine position mouse experiment. **a** Radioluminescent image of the mouse. **b** 3D fusion image of CT and reconstructed radioluminescence signals. **c** Transversal, coronal and sagittal slices of reconstructed radioluminescence signals overlaid on corresponding CT slices

For artificial source-implanted mouse model experiment, the animal was cared for in accordance with a protocol approved by the Fourth Military Medical University Animal Care and Use Committee. A Kunming mouse with abdomen unhaired by depilatory cream was used as the imaged animal. A pseudo tumor was provided by injecting the mixture of 100 µL Na^99^mTcO_4_ with an activity 3.7 MBq (100 µCi), 10 mg Gd_2_O_2_S:Tb and 100 µL matrigel (BD Biosciences, Sparks, MD) into right abdomen of the mouse under general anesthesia by inhalation of 1–2% isoflurane-oxygen. Radioluminescent image and CT projection images of the mouse were acquired using the same parameter settings and procedures as those in the former phantom experiment. There is obvious radioluminescence at the pseudo tumor location with low background noises (Fig. 5a). After getting the surface of the mouse based on CT reconstruction, we constructed a homogeneous diffuse model with absorption and scattering coefficients of 0.002 and 1 mm^−1^, respectively.

The results of artificial source-implanted mouse model experiment are summarized in Fig. 5, and the 3D volume rendering of GRLT reconstruction is in accordance with CT image (Fig. 5b). Transversal, coronal and sagittal slices of GRLT are shown in Fig. 5c. This result shows the ability of GRLT to recover the RLNPs distributions in biology tissues for a mouse model.

The Cerenkov luminescence is emitted when charged particles generated form radioactive decay of radiotracer propagating in biology tissues. So CLI reveals the distribution of radiotracer, which is similar with PET or SPECT. While in RELI, radiotracer just serves as an internal excitation source to excite RLNPs, and its imaging target is RLNPs but not radiotracer. Cerenkov luminescence is much weaker than that of RELI, so RELI have much higher sensitivity and signal to noise ratio than those of CLT when tumors have a low uptake of radiotracer, which is helpful for GRLT to detect tumors at early stage or with a low metabolism. However the Gamma rays excited radioluminescence is rather weak compared to fluorescence of many fluorescent probes. To get high enough signal, we used a high concentration of the RLNPs in the artificial source-implanted mouse model experiment. Some brighter RLNPs are helpful for this method in practical application. Another limitation is the higher radiation dose to the subject, due to the long half-life time of radionuclide ^99m^Tc.

GRLT is an extension of RELI from two dimensions to three dimensions, which can provide distribution of RLNPs deep in biology tissues. This may provide a useful optical method for imaging deep tumors in preclinical studies. Noting that there is no penetration limitation of the internal excitation source due to the excellent penetration ability of gamma rays, but the peak wavelength of emitted radioluminescence for Gd_2_O_2_S:Tb is 545 nm, which has a poor penetration depth. Using near infrared spectral region RLNPs such as Gd_2_O_2_S:Eu may improve the imaging depth of GRLT.

This work is a preliminary study of tomographic imaging based on radioluminescence. The experiments have simplified the complex distribution of radiotracer by using a mixture of RLNPs and radiotracers. And the three-dimensional quantification of RLNPs has not been realized in this work, for one thing, we have not acquired water soluble RLNPs, for another thing, how to accurately obtain the distribution of radiotracer should be solved firstly. In the future work, we will try to overcome these problems and perfect this method.

In the artificial source-implanted mouse experiment, we injected the mixture of RLNPs with Na^99m^TcO_4_ into the mouse, which is different with a real in vivo experiment, because the physiological uptake in a tumor and the background are neglected. In practice we can use targeting RLNPs to trace tumor, and radionuclide to excite the targeted RLNPs.

## Conclusion

In conclusion, results of phantom and artificial source-implanted mouse experiments have demonstrated the ability of GRLT to reveal the distribution of RLNPs such as Gd_2_O_2_S:Tb using the radioluminescent signals when excited by gamma rays produced from ^99m^Tc. With the emerging of targeted RLNPs, we believe GRLT can provide new possibilities for in vivo and noninvasive examination of biological processes at cellular levels. Especially, combining with Cerenkov luminescence imaging, GRLT can achieve dual molecular information of RLNPs and nuclides using single optical imaging technology.

## Abbreviations

RELI: radionuclide-excited luminescence imaging
RLNP: radioluminescent nanophosphor
GRLT: gamma rays excited radioluminescence tomography
CLT: Cerenkov luminescence imaging
ROI: region of interesting
REFT: radiopharmaceutical excited fluorescence tomography
